# Knowledge, care-seeking behavior, and factors associated with patient delay among newly-diagnosed pulmonary tuberculosis patients, Federal Capital Territory, Nigeria, 2010

**DOI:** 10.11694/pamj.supp.2014.18.1.4166

**Published:** 2014-07-21

**Authors:** Oladayo Biya, Saheed Gidado, Ajibola Abraham, Ndadilnasiya Waziri, Patrick Nguku, Peter Nsubuga, Idris Suleman, Akin Oyemakinde, Abdulsalami Nasidi, Kabir Sabitu

**Affiliations:** 1 Nigeria Field Epidemiology and Laboratory Training Program, Abuja, Nigeria; 2 Global Public Health Solutions, Decatur, Georgia, United States of America; 3 Ahmadu Bello University, Zaria, Nigeria; 4 Nigeria Centre for Disease Control, Federal Ministry of Health, Abuja, Nigeria

**Keywords:** Directly observed treatment short course, Tuberculosis, patient delay, Federal Capital Territory, Nigeria

## Abstract

**Introduction:**

Early treatment of Tuberculosis (TB) cases is important for reducing transmission, morbidity and mortality associated with TB. In 2007, Federal Capital Territory (FCT), Nigeria recorded low TB case detection rate (CDR) of 9% which implied that many TB cases were undetected. We assessed the knowledge, care-seeking behavior, and factors associated with patient delay among pulmonary TB patients in FCT.

**Methods:**

We enrolled 160 newly-diagnosed pulmonary TB patients in six directly observed treatment short course (DOTS) hospitals in FCT in a cross-sectional study. We used a structured questionnaire to collect data on socio-demographic variables, knowledge of TB, and care-seeking behavior. Patient delay was defined as > 4 weeks between onset of cough and first hospital contact.

**Results:**

Mean age was 32.8 years (± 9 years). Sixty two percent were males. Forty seven percent first sought care in a government hospital, 26% with a patent medicine vendor and 22% in a private hospital. Forty one percent had unsatisfactory knowledge of TB. Forty two percent had patient delay. Having unsatisfactory knowledge of TB (p = 0.046) and multiple care-seeking (p = 0.02) were significantly associated with patient delay. After controlling for travel time and age, multiple care-seeking was independently associated with patient delay (Adjusted Odds Ratio = 2.18, 95% CI = 1.09-4.35).

**Conclusion:**

Failure to immediately seek care in DOTS centers and having unsatisfactory knowledge of TB are factors contributing to patient delay. Strategies that promote early care-seeking in DOTS centers and sustained awareness on TB should be implemented in FCT.

## Introduction

Tuberculosis (TB) is a major cause of illness and death worldwide, especially in Asia and Africa. Globally, 9.2 million new cases and 1.7 million deaths from TB occurred in 2006 [1]. Nigeria ranked fourth globally and first in Africa among World Health Organization's (WHO's) list of 22 TB high-burden countries [2]. In 2007, Nigeria had an estimated TB incidence rate of 311 cases per 100,000 population per year and a TB mortality rate of 93 deaths per 100,000 population per year [2]. In 1994, WHO introduced directly observed treatment short course (DOTS) as a global strategy to reduce TB burden [3]. The DOTS strategy recommends detecting 70% of TB cases. By the end of 2008, DOTS services attained 100% coverage of all 774 Local Government Areas (LGAs) in Nigeria [4]. In line with recommendations of WHO, TB case detection in Nigeria is based on passive case finding using sputum-smear microscopy. This requires TB patients seek an adequate health facility for diagnosis and treatment. The tendency and urgency of TB patients to seek care may be influenced by their knowledge of the disease and the perceived risk it poses to individuals, families, and communities at large [5]. It is important to identify and treat TB cases as early as possible because each day that an open case of TB goes untreated, the risk of infecting others increases [6]. However, studies have shown that many TB patients adopt multiple care-seeking before consulting a healthcare facility [7, 8]. Delay in presentation to a health facility contributes to delays in initiating TB treatment, and can result in greater morbidity and mortality for the patient [9–13]. Human Immunodeficiency Virus (HIV) infection, low access to healthcare, and attendant delay in care-seeking contribute to the persistent burden of TB [14]. The Federal Capital Territory (FCT) Nigeria combines a low TB Case Detection Rate (CDR) with a high HIV prevalence. In 2007, FCT reported a CDR of 9%, the lowest in the country, and had HIV prevalence of 9.9%, the third highest in the country [15, 16]. In FCT, no study has assessed the knowledge and perceptions of TB patients about the disease. TB control cannot be effective unless erroneous beliefs among people are identified and corrected [17]. Better understanding of the places where TB patients first seek care and factors responsible for delay is important to improve TB case finding and institute early treatment. This study was conducted to assess the knowledge, care-seeking behavior, and factors associated with patient delay among TB patients in FCT.

## Methods

**Study Setting:** We conducted the study in six hospitals in FCT, the capital of Nigeria. The projected population for FCT for 2010 was 2.0 million people, based on the 2006 national population census. The hospitals where the study was conducted are University of Abuja Teaching Hospital (UATH) Gwagwalada, and Maitama, Asokoro, Nyanya, Bwari, and Kuje General Hospitals. UATH Gwagwalada is a tertiary referral hospital for FCT and adjoining Kogi and Niger States while the remaining five hospitals are secondary hospitals that serve mainly people from FCT. All the hospitals provide free TB diagnosis and treatment under the coordination of Nigeria's National TB and Leprosy Control Programme (NTBLCP). An average of 12 new pulmonary TB patients are diagnosed in a month in UATH, Gwagwalada, eight for each of Maitama and Asokoro General Hospitals (GH), seven for Nyanya GH, three for Bwari GH and two for Kuje GH.

**Study Design:** We conducted a cross sectional study from March to June 2010. Newly-diagnosed pulmonary TB patients were recruited consecutively and interviewed within 1 week of commencement of anti-TB treatment. Diagnosis was by sputum-smear microscopy. The patients were recruited from secondary and tertiary hospitals offering both diagnostic and treatment services for DOTS in FCT. Primary-level hospitals were not included because of the very few patients that would be obtained within the 4 months scheduled for the study.

**Sample Size Determination:** The calculation of the sample size was based on a hypothesized proportion (p) of TB patients who present early of 17% [18], significance level of 5% -corresponding to a standard normal deviate (z) of 1.96, power of 80%, and precision (d) of 6%. We used the formula: n = z2pq/d2 and obtained a minimum sample size (n) of 151 children. Adding 5% for non-usable data, we sampled a total of 160 TB patients.

**Sampling Technique:** A stratified sampling technique was used to select the study sites. All the 12 hospitals with DOTS services in FCT were stratified into secondary and tertiary hospitals. Out of the two tertiary hospitals, one was selected by random sampling, which was the UATH Gwagwalada. Of the 10 secondary hospitals, five (Maitama, Asokoro, Nyanya, Bwari and Kuje GH) were selected by random sampling. Patients were allocated to individual study sites proportionate to the average number of new pulmonary TB patients diagnosed in a month. Based on this, we recruited 48 patients from UATH Gwagwalada, 32 patients from each of Maitama and Asokoro GH, 28 patients from Nyanya GH, 12 patients from Bwari GH and eight patients from Kuje GH.

**Data Collection and Analysis:** We used a structured questionnaire to collect data on socio-demographic characteristics, knowledge and care-seeking behavior. Three nurses were trained and used as interviewers. The questionnaire instrument was pre-tested in a secondary DOTS hospital in FCT that was not used as a study site. The questionnaire was administered on newly-diagnosed pulmonary TB patients in the study sites within the study period. We excluded patients less than 15 years, extra-pulmonary TB, category 2 TB (e.g., relapse, treatment failure, or return after default) and those who denied consent. Data obtained were analyzed using Epi Info version 3.5.1. Descriptive and analytical statistics were used to summarize the data. Descriptive statistics involved use of frequencies, proportions, tables and graphs. Analytical statistics through bivariate analysis and stepwise logistic regression were performed to identify factors associated with patient delay in treatment. For bivariate analysis, odds ratios were measured to identify associations while 95% confidence interval (CI) and p values were used to determine statistical significance. For stepwise logistic regression, all factors with p values < 0.1 in the bivariate analysis were included in the logistic regression model. Adjusted odds ratios (AORs) were determined with 95% confidence interval (CI) to identify independent factors. Seven questions were used to assess knowledge on TB. These questions were on who a suspected case of TB is, cause of TB, mode of transmission of TB, relationship between TB and HIV, need for screening of household members, duration of TB treatment, and awareness on drug-resistant TB. Answering five or more questions correctly was regarded as satisfactory knowledge. In this study, we defined patient delay as period more than 4 weeks from onset of symptoms to first contact with a hospital [18]. Multiple care-seeking was defined as seeking care in two or more non-DOTS centers before reaching the DOTS hospital where patient was interviewed. A patent medicine vendor (PMV) was defined as a person without formal pharmacy training who sells pharmaceutical products on a retail basis for profit.

**Ethical Consideration:** The study was approved by FCT Health Research Ethics Committee. Informed consent was obtained from each respondent.

## Results

### Socio-demographic Characteristics

The mean age was 32.8 years (standard deviation ± 9.0 years). Sixty two percent were males, 54% had secondary-level education, while 76% lived within a 20 minutes travel time to the nearest health facility. The occupation, marital status and religion of the patients are shown in Table 1.

**Table 1:** Socio-demographic Characteristics of Newly-diagnosed Pulmonary Tuberculosis Patients, Federal Capital Territory, Nigeria, 2010

Variable	Frequency (n = 160)	Proportion (%)
**Age group**		
15 – 24	27	16.9
25 – 34	71	44.4
35 – 44	41	25.6
≥ 45 years	21	13.1
**Gender**		
Male	99	61.9
Female	61	38.1
**Occupation**		
Formal	54	33.8
Informal	57	35.6
Student	26	16.3
Unemployed	23	14.2
**Level of education**		
No formal education	9	5.6
Quranic	2	1.3
Primary	34	21.3
Secondary	87	54.4
Post-secondary	28	17.5
**Marital Status**		
Single	75	46.9
Married	80	50.0
Divorced	3	1.9
Widowed	2	1.3
**Religion**		
Christianity	127	79.4
Islam	33	20.6
**Travel time (minutes)**		
≤ 20	122	76.3
> 20	38	23.7

### Knowledge of Tuberculosis

Eighty four percent were aware that cough is a symptom of pulmonary TB, but were less aware of the other symptoms (Table 2). On the cause of TB, 36% knew that TB is caused by a germ. Fifty nine percent knew that TB could be transmitted airborne while 3% knew that drinking infected cow's milk could transmit TB. On factors that could increase TB transmission, 31% were aware of overcrowding while 11% were aware of depressed immunity. Ninety eight percent knew that TB is treated over a period of 8 months and 68% felt there was need for screening of household members. The main source of information about TB was health worker for 61% while 15% heard from each of friend/relative and news media. Based on the response to the seven questions used to assess knowledge, 41% had satisfactory knowledge (Table 2). There was no significant association between level of education and knowledge (p = 0.20).

**Table 2:** Knowledge of Newly-diagnosed Pulmonary Tuberculosis Patients on the Disease, Federal Capital Territory, Nigeria, 2010

Variable	Frequency (n = 160)	Proportion (%)
**Symptoms of TB**		
Cough	134	83.8
Chest pain	44	27.5
Fever	40	25.0
Weight loss	38	23.8
Body weakness	38	23.8
Night sweats	20	12.5
Hemoptysis	11	6.9

### Care-seeking Behavior

Forty seven percent first sought care from government hospital while 26% and 22% first sought care from PMVs and private hospitals respectively. Two percent first sought care from each of religious house and traditional healer. Thirty three percent sought care at multiple (range 2 -5, median 3) sites before going to a DOTS hospital where they were interviewed.

### Determinants of Patient Delay

The median time from onset of illness to first hospital contact was 4 weeks (range: 1 -30 weeks). Patient delay was observed in 42% patients. Multiple care-seeking and unsatisfactory knowledge were significantly associated with patient delay (p < 0.05). There was no significant association between level of education and age with patient delay (Table 3). After controlling for age and travel time from residence to nearest hospital, multiple care-seeking was significantly associated with patient delay (AOR = 2.18, 95% CI = 1.09-4.35) (Table 4).

**Table 3:** Factors Associated with Patient Delay among Newly-diagnosed Pulmonary Tuberculosis Patients, Federal Capital Territory, Nigeria, 2010

Variable	Patient delay	No patient delay	Odds ratio	95% Confidence interval
**Multiple care-seeking**				
Yes	29	23	2.32	1.12 – 4.83
No	38	70		
**Knowledge level**				
Unsatisfactory	46	48	2.05	1.01 – 4.18
Satisfactory	21	45		
**Travel time (minutes)**				
> 20	17	21	1.17	0.56 – 2.43
≤ 20	50	72		
**Sex**				
Male	38	61	0.69	0.36 – 1.31
Female	29	32		
**Age**				
15-35	41	66	0.65	0.33 – 1.25
> 35	26	27		
**Marital status**				
Single/Divorced/Widowed	33	47	0.95	0.50 – 1.78
Married	34	46		
**Level of education**				
None/Quranic/Primary	21	24	1.31	0.66 – 2.62
Secondary/tertiary	46	69		

**Table 4:** Independent Factors Associated with Patient Delay among Pulmonary Tuberculosis Patients in Federal Capital Territory, Nigeria, 2010

Variable	Adjusted Odds ratio	95% C.I
Multiple care-seeking	2.18	1.09 – 4.35
Unsatisfactory knowledge	1.95	0.94 – 3.82
Travel time > 20 minutes	1.08	0.77 – 3.18
Age	0.74	0.37 – 1.50

**NB:** These results are based on a logistic model that includes multiple care-seeking, knowledge level, travel time and age

## Discussion

We found limited knowledge about TB and multiple care-seeking efforts among newly-diagnosed pulmonary TB patients in FCT in 2010. Patients who had unsatisfactory knowledge and those with multiple care-seeking were more likely to delay in seeking care. In our study, 41% of TB patients had satisfactory knowledge. This level of knowledge is low and comparable to the 30% in Tanzania [19] but much lower than 64% in Iraq [20]. However, the higher level of knowledge in the Iraq study could possibly be explained by the higher level of education among the patients. The association between knowledge and level of education has been further shown in studies in Tanzania [19] and Vietnam [21] but this was not demonstrated in our study. Also, 40% did not know that TB could be transmitted airborne. This low awareness on mode of transmission among TB patients has been documented in other studies [22–24]. Ignorance on how TB is transmitted could be a reason why these patients got infected because they likely failed to adopt preventive measures. Even though occurrence of M. bovis infection in humans has largely reduced globally following introduction of pasteurization of milk, transmission of TB through drinking infected cow's milk still occurs. However, our study showed low awareness on this mode of TB transmission. The implication of this is that the common practice of drinking unpasteurized milk called “nunu” in FCT and other parts of northern Nigeria may be exposing many inhabitants to M. bovis infection. Closely related to the issue of transmission of TB is the need for screening of household members. A TB patient needs to know that people living close to him or her are at risk of getting the disease or may already have latent infection. Up to a quarter of TB patients did not see the need for household members to be screened for TB disease, which could indicate that they did not understand the risk they posed to people living around them [25].

Health workers were the main source of TB information similar to what was previously documented in Tanzania [19]. This finding may be because health workers in DOTS hospitals typically counsel TB patients before commencement of treatment. In contrast, 15% of respondents cited the mass media as their main source of information on TB, as was observed in a study in Iraq [20]. This low proportion could suggest inadequate coverage on TB through the mass media, a channel of communication that has potential to reach several people at the same time. However, health education alone through the mass media may not be enough to reach and influence all potential TB cases. Also, 15% of the respondents obtained information on TB from friends and relatives. The implication of this is that if some patients depend on information that friends and relatives give, there would be need to sensitize communities on TB to prevent misinformation.

In this study, 27% of TB patients first sought for care from non-medical facilities, similar to findings from Lagos, Nigeria [18] but higher than for Malawi [26]. This proportion is high considering the fact that there has been 100% coverage for DOTS services in Nigeria since 2008 [4]. Among the non-medical facilities, PMVs were the most sought place for initial care as documented in other studies [27, 28]. This high patronage of PMVs may be related to the general practice of purchase of over-the-counter drugs for common ailments in Nigeria. Based on this finding, there is need to involve PMVs in the referral chain of TB patients to DOTS centers in order to improve case finding. It is likely that the communities do rely on PMVs as their first point of care for many health-related complaints, making it imperative to involve them in the fight against TB. Among those who first presented in medical facilities, more patients sought care in a government hospital unlike in the study in Lagos, Nigeria [18] where more patients presented at private hospitals. It is however known that Lagos is an economic hub and has one of the highest concentrations of private hospitals in Nigeria [18]. Despite this, one-fifth of TB patients in our study first presented in a private hospital, the majority of which use shorter treatment regimens outside the recommendations of NTBLCP [29]. This has important public health implications as drug-resistant TB is a possible sequelae in improperly-treated TB patients [27]. The median time from onset of illness to first hospital contact was 4 weeks. This is consistent with findings from Argentina, Norway, and Queensland Australia [30–32] but lower than 8 weeks reported in Lagos, Nigeria [18]. The prevalence of patient delay was found to be high at 42%, similar to findings from Ondo, Nigeria [22]. Determinants of patient delay were having unsatisfactory knowledge of TB and multiple care-seeking before going to the DOTS center. The association of low level of knowledge of TB with patient delay has been documented in other studies [27, 33]. Also, the association of preceding multiple care-seeking with patient delay is consistent with findings from India [7]. It can be explained that a patient who visits a PMV or other unorthodox places would have wasted ample time before reaching the right place for treatment. The multiple care-seeking pattern of one third of our respondents is similar to findings from Enugu, Nigeria [27].

The interpretation of the findings of our study is subject to some limitations. The first is that respondents were those that eventually sought care in a public DOTS center. Their characteristics and views may be different from those that never sought care in these centers, so the findings cannot be generalized to the total population of TB patients in FCT. Secondly, patient delay in care-seeking was recorded based on patient's recall ability. However, we recruited and interviewed our respondents within 1 week of commencement of anti-TB treatment to minimize recall bias.

## Conclusion

PMVs and private hospitals are among the major places where TB patients first seek care following onset of illness. Multiple and delayed care-seeking were found in many patients before they reported to the DOTS center. Patient delay was associated with having unsatisfactory knowledge of TB and multiple care-seeking before reporting at the DOTS center. FCT TB Control Program should organize sensitization programs on TB for PMVs and owners of private hospitals to increase their understanding of TB and ensure that they refer suspected TB cases promptly to DOTS centers where they can be diagnosed and properly treated.

## References

[1] World Health Organization (2008). Global tuberculosis control -surveillance, planning, financing.

[2] World Health Organization (2009). Global tuberculosis control-epidemiology, strategy, financing.

[3] World Health Organization (1994). Framework for effective tuberculosis control. WHO Tuberculosis Programme.

[4] National Tuberculosis and Leprosy Control Programme (2008). Federal Ministry of Health, Abuja, Nigeria.

[5] Alvarez Gordillo (2001). Seeking tuberculosis care in Chiapas, Mexico. Revista Panamericana de Salud Pública..

[6] Sadiq H, Muynck AD (2001). Health care seeking behavior of pulmonary tuberculosis patients visiting TB center Rawalpindi. J Park Med Assoc..

[7] Pathania Vikram, Almeida Joel, Kochi Arata (1997). Tuberculosis patients and private for profit health care providers in India. Global TB Programme of the World Health Organization.

[8] Auer Christian, Sarol Jesus, Tanner Marcel, Weiss Mitchell (2000). Health-seeking and perceived causes of tuberculosis among patients in Manilla, Philippines. Trop Med Int Health.

[9] Barker RD, Millard FJ, Malatsi J (2006). Traditional healers, treatment delay, performance status and death from tuberculosis in rural South Africa. Int J Tuberc Lung Dis..

[10] Golub JE, Bur S, Cronin WA (2006). Delayed tuberculosis diagnosis and tuberculosis transmission. Int J Tuberc Lung Dis..

[11] Lin Xu, Chongsuvivatwong Virasakgi, Lin Lu, Geater Alan, Lijuan Ren (2008). Dose-response relationship between treatment delay of smear-positive tuberculosis patients and intra-household transmission: a cross-sectional study. Trans R Soc Trop Med Hyg..

[12] Madebo T, Lindtjorn B (1999). Delay in Treatment of Pulmonary Tuberculosis: An Analysis of Symptom Duration Among Ethiopian Patients. Med Gen Med.

[13] Nair Dinesh, George Annie, Chacko K (1997). Tuberculosis in Bombay: new insights from poor urban patients. Health Policy and Planning..

[14] Mesfin Mengiste, Tesfay Tasew, Israel Tareke, Madeley Richard (2005). Delays and care-seeking behavior among Tuberculosis patients in Tigray of northern Ethiopia. Ethiop J Health Dev..

[15] National Tuberculosis and Leprosy Control Program (2007). Federal Ministry of Health, Abuja, Nigeria.

[16] National AIDS and STDs Control Program National HIV/AIDS Seroprevalence Sentinel Survey Report. 2008.

[17] Khan Amir, Walley John, Newell James, Imdad Naghma (2000). Tuberculosis in Pakistan: Socio-Cultural Constraints and opportunities in treatment. Soc Sci Med..

[18] Olumuyiwa Odusanya, Babafemi Joseph (2004). Patterns of delays amongst pulmonary tuberculosis patients in Lagos. BMC Public Health..

[19] Wandwalo ER, Morkve O (2000). Knowledge of disease and treatment among tuberculosis patients in Mwanza, Tanzania. Int J Tuberc Lung Dis..

[20] Nguyen Hoa, Anna Thorson, Vinod Diwan (2003). Knowledge of tuberculosis and associated health-seeking behaviour among rural Vietnamese adults with a cough for at least three weeks. Scand J Public Health..

[21] Hashim DS, Al Kubaisy W, Al Dulayme A (2003). Knowledge, attitudes and practices survey among health care workers and tuberculosis patients in Iraq. Eastern Mediterranean Health Journal..

[22] International Center for Gender and Social Research (2008). National Tuberculosis Survey: Report of the knowledge, attitude, behavior, experiences and practices in Southeast.

[23] Mohammed AI, Yousif MA, Ottoa P (2007). Knowledge of Tuberculosis: A survey among Tuberculosis patients in Omdurman, Sudan. Sudanese Journal of Public Health..

[24] Bhat S, Singal N, Aggarwal CS, Jain RC (1999). Knowledge attitudes and practices of newly diagnosed sputum positive cases of pulmonary tuberculosis. Journal of Communicable Diseases..

[25] Liam CK, Lim KH, Wong CMM, Tang BG (1999). Attitudes and knowledge of newly diagnosed tuberculosis patients regarding the disease, and factors affecting treatment compliance, Malaysia. Int J Tuberc Lung Dis..

[26] Salaniponi FM, Harries AD, Banda HT, Kang'ombe C (2000). Care seeking behaviour and diagnostic processes in patients with smear-positive pulmonary tuberculosis in Malawi. Int J Tuberc Lung Dis..

[27] Okeibunor JC, Onyeneho NG, Chukwu JN, Post E (2005). Where do Tuberculosis patients go for treatment before reporting to DOTS clinics in Southern Nigeria?. A study report submitted to the German Leprosy and Tuberculosis Relief Association, Enugu, Nigeria.

[28] Kiwuwa Mpungu, Charles Karamagi, Harriet Mayanja (2005). Patient and health service delay in pulmonary tuberculosis patients attending a referral hospital: a cross-sectional study. BMC Public Health..

[29] Alakpa GE, Edet EA (1999). Management of pulmonary tuberculosis PTB: regimen employed in Lagos, Nigeria. West Afr J Med..

[30] Zerbini E, Chirico M, Salvadores B (2008). Delay in Tuberculosis diagnosis and treatment in four provinces of Argentina. Int J Tuberc Lung Dis..

[31] Farah Mohamed, Rygh Jens, Steen Tore, Gunna Bjune (2006). Patient and health care system delays in the start of Tuberculosis treatment in Norway. BMC Infect Dis..

[32] Ward J, Siskind V, Konstantinos A (2001). Patient and health care system delays in Queensland Tuberculosis patients, 1985-1998. Int J Tuberc Lung Dis..

[33] World Health Organization (2006). Diagnostic and treatment delay in Tuberculosis -An in-depth analysis of the health-seeking behavior of patients and health system response in seven countries of the Eastern Mediterranean Region.

